# Data on the power of high betatrophin to predict cardiovascular deaths in coronary patients

**DOI:** 10.1016/j.dib.2019.104989

**Published:** 2019-12-12

**Authors:** Andreas Leiherer, Janine Ebner, Axel Muendlein, Eva M. Brandtner, Christina Zach, Kathrin Geiger, Peter Fraunberger, Heinz Drexel

**Affiliations:** a Vorarlberg Institute for Vascular Investigation and Treatment (VIVIT), Feldkirch, Austria; b Private University of the Principality of Liechtenstein, Triesen, Liechtenstein; c Medical Central Laboratories, Feldkirch, Austria; d Drexel University College of Medicine, Philadelphia, PA, USA; e Division of Angiology, Swiss Cardiovascular Center, University Hospital of Bern, Switzerland

**Keywords:** Betatrophin, ANGPTL8, Lipasin, Cardiovascular death, Biomarker, Cardiovascular events, Coronary patients

## Abstract

Betatrophin is a protein which is produced by the liver and by adipose tissue. There are no clear data about serum betatrophin's cardiovascular role and it is unknown, whether betatrophin is associated with the risk of cardiovascular death. This article provides additional data on the association of betatrophin with its power to predict cardiovascular death in coronary patients. In addition, this data article demonstrates the performance of betatrophin as a biomarker using c-statistics. Analyzed data was derived from 553 coronary patients. Betatrophin was measured in serum samples and cardiovascular deaths were recorded for a median of 7.1 years. This data article is related to a research article titled “High betatrophin in coronary patients protects from cardiovascular events” [1].

**Table:** Specifications table

Subject area	*Medicine, Clinical Research*
More specific subject area	*Cardiology, Epidemiology, Biomarkers*
Type of data	*Figures, table*
How data was acquired	*ELISA*
Data format	*Raw data and analyzed data*
Experimental factors	*Betatrophin concentration of 553 coronary patients has been determined and cardiovascular deaths have been recorded for a median of 7.1 years*
Experimental features	*Betatrophin in serum samples was measured by ELISA*
Data source location	*Feldkirch, Austria*
Data accessibility	*Analyzed data is with this article. Raw data is available upon request and approval (Contact:*vivit@lkhf.at*).*
Related research article	*Leiherer, A., Ebner, J., Muendlein, A, Brandtner, E., Zach, C. Geiger, K., Fraunberger, P., and Drexel, H., 2019. High betatrophin in coronary patients protects from cardiovascular events. Atherosclerosis.*

**Table:** 

**Value of the data** - No prospective data on the power of betatrophin to predict the cardiovascular risk were available at present. - Whereas the main article “High betatrophin in coronary patients protects from cardiovascular events” has evaluated the performance of uromodulin as a biomarker to predict cardiovascular events, this data article is focused on cardiovascular mortality. - This data article helps researchers to evaluate the potential of betatrophin as a cardiovascular biomarker over and above the main article. - These data are important, because the role of betatrophin in cardiovascular disease is still elusive, and may stimulate future research on betatrophin.

## 1 Data

**Fig. 1:**
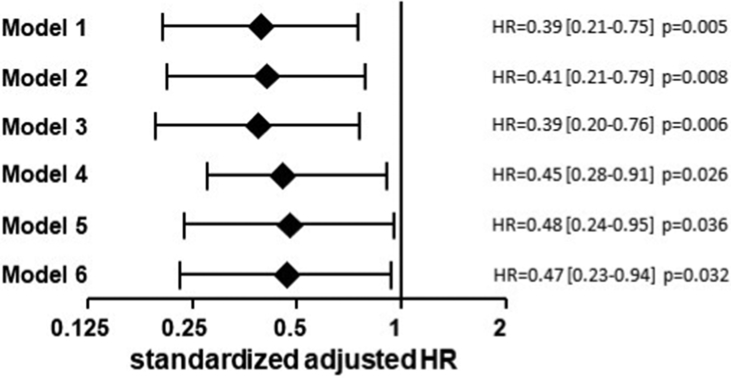
**Betatrophin as predictor of cardiovascular death.** The Forest plot represents the hazard ratios (HR) with 95% confidence interval (CI) for the association between high betatrophin (status) in serum and cardiovascular death risk in the study population. Model 1 represents a univariate analysis. Model 2 includes the covariates age, gender, and body mass index (BMI). Model 3 includes the parameters included in model 2 and in addition the concentration of triglycerides, of high density lipoprotein (HDL) and low density lipoprotein (LDL) cholesterol, and the type 2 diabetes (T2DM), hypertension, current smoking and significant coronary artery disease (CAD) status. Model 4 includes the parameters included in model 3 and in addition the Vitamin D concentration. Model 5 includes the parameters included in model 4 and in addition the estimated glomerular filtration rate (eGFR) and the concentration of brain natriuretic peptide (BNP), C-reactive protein (CRP), and troponin I. Model 6 includes the parameters included in model 5 and in addition the treatment status with respect to acetylsalicylic acid (ASA), beta blocker, angiotensin converting enzyme (ACE) inhibitors, angiotensin (AT)-2 receptor antagonists, and statins.

It has been mentioned in the main article [1] that there is a significant association between high betatrophin in serum of 553 coronary patients and a low risk for cardiovascular events. Here, further data on the association between betatrophin and cardiovascular death are provided. In total 64 patients succumbed to cardiovascular death. Table 1 gives an overview about the frequency of different types of cardiovascular deaths. There were no significant differences between different groups (p = 0.768) nor between vascular-related (vascular death + fatal insult) and cardiac-related (fatal myocardial infarction, sudden cardiac death, terminal heart failure, and other cardiac death) deaths (p = 0.692). Fig. 1 demonstrates that high betatrophin protects from cardiovascular death during follow up time. The data summarized in Table 2 shows the comparison of different prediction models for cardiovascular death. The prediction of cardiovascular deaths is significantly higher applying an enhanced prediction model comprising betatrophin compared to a basic model lacking betatrophin.

**Table 1:** Causes of cardiovascular deaths.

Cause	n	Betatrophin, mean ± SD (ng/ml)
Vascular death	5	8.2 ± 2.9
Fatal insult	8	6.8 ± 1.9
Fatal myocardial infarction	5	6.6 ± 1.5
Sudden cardiac death	3	4.9 ± 0.9
Terminal heart failure	21	8.4 ± 7.2
Other/unknown cardiac death	22	15.7 ± 28.8
total	64	10.4 ± 17.6

Sixty-four cardiovascular deaths were recorded during follow up. Betatrophin concentration is given as mean ± standard deviation (SD).

**Table 2:** Biomarker evaluation for cardiovascular death data in prediction models with and without betatrophin.

Outcome	Model	AUC	Harrell's C	Somers’D	IDI NRI	p-value
CV death	default	0.771	0.757	0.515	0.070	0.003
	default + betatrophin	0.780	0.768	0.536	0.764	<0.001
CV death	SCORE	0.717	0.625	0.250	0.068	0.002
	SCORE + Betatrophin	0.732	0.665	0.330	0.794	<0.001

Betatrophin's ability to improve risk stratification for cardiovascular death is analyzed by comparing a default cardiovascular risk model (“default”) comprising continuous variables age, sex, BMI, LDL, HDL, triglycerides, and categorical variables hypertension status, the current smoking status, the diabetes mellitus type 2 status, and the significant CAD status to a model additionally comprising the betatrophin status as a categorical variable (“default + Betatrophin”). Similarly, the ESC-proposed SCORE algorithm model (“SCORE”) comprising continuous variables age, sex, total cholesterol, systolic blood pressure, and the categorical variable current smoking status was compared to the SCORE model comprising all mentioned variables and in addition betatrophin status as a categorical variable (SCORE+Betatrophin”). Models were built as linear predictor scores after Cox regression. Harrell’s C, Somers’ D and the area under the curve (AUC) for the receiver operator characteristic (ROC) are given. Integrated discrimination improvement (IDI) and net reclassification improvement (NRI) indices for the addition of betatrophin to the basic model are given with respective p-values at follow up end.

## 2 Experimental design, materials and methods

### 2.1 Design and analyses

Characterization and basic laboratory measurement of patients was done as described in the main article [1]. In short, all 553 coronary patients were recruited between 2005 and 2008. Only patients who were referred to elective coronary angiography for the evaluation of established or suspected stable CAD were enrolled. Patients undergoing coronary angiography for other reasons and patients with acute coronary syndromes were excluded. Serum betatrophin levels were determined with a commercial uromodulin enzyme-linked immunosorbent assay (ELISA) kit (BioVendor, Brno, Czech Republic; catalog no. RD191347200R), which was specific for human betatrophin. The inter- and intra-assay coefficient of variation was ≤3.9% and 7.4% respectively. Serum betatrophin was 10.4 ± 12.9 ng/ml (mean ± SD) on average and its median was 7.5 ng/ml with an IQR of 5.4–10.6 ng/ml and minimum (min) and maximum (max) of 1.2 ng/ml and 136.4 ng/ml respectively.

The median follow up time of the study, described in the main article, was 7.1 years with an interquartile range (IQR) of 5.9–7.5 years. No patients were lost during follow up, resulting in a 100% follow-up rate. The endpoint “cardiovascular death” was a composite of fatal insult, vascular death, fatal myocardial infarction, sudden cardiac death, terminal heart failure, and other cardiac death.

### 2.2 Statistical analysis

Statistical analyses are described in detail in the main article [1]. In particular, to determine the incidence of the respective endpoint, we used Cox proportional hazards models. In the case continuous data showed a skewed distribution (skewness >1 or < -1), these data were log transformed and z-transformed before analysis. In order to examine the value of uromodulin as a biomarker [2] several cox regression models were fitted with the respective study endpoint as the dependent variable and c-statistics were applied. The respective models were compared according to their linear predictor score using calculation of area under the curve (AUC), Harrell's C and Somers' D, as well as integrated discrimination improvement (IDI) and net reclassification improvement (NRI) indices. All missing values were missing completely at random (MCAR), according to Little's MCAR test. All data were analyzed according to complete case analysis. All statistical analyses as described in this data article were performed with SPSS 26.0 for Windows (SPSS, Inc., Chicago, IL) and R statistical software v. 3.6.1 (http://www.r-project.org) including specific software packages (survIDINRI [3,4]).
